# Poly[bis­(acetone-κ*O*)bis­{μ_3_-1-[(5-chloro-2-oxidophenyl)diazenyl]-2-naphtholato-κ^4^ *O*:*O*,*O*′:*O*′}­sodium(I)­chromium(III)]

**DOI:** 10.1107/S1600536808000433

**Published:** 2008-01-11

**Authors:** Shinpei Ito, Yohei Sato, Jin Mizuguchi

**Affiliations:** a Department of Applied Physics, Faculty of Engineering, Yokohama National University, Tokiwadai 79-5, Hodogaya-ku, Yokohama 240-8501, Japan; b Department of Applied Physics, Graduate School of Engineering, Yokohama National University, Tokiwadai 79-5, Hodogaya-ku, Yokohama 240-8501, Japan

## Abstract

The title compound, [CrNa(C_16_H_9_ClN_2_O_2_)_2_(C_3_H_6_O)_2_]_*n*_, is an azo-Cr^III^ complex polymer that is used as a charge-control agent in electrophotography. The monomeric unit is composed of octa­hedral Cr^III^ and Na^I^ units, and is characterized by twofold rotation symmetry. The Cr^III^ atom is chelated by two N and four O atoms from two [(5-chloro-2-oxidophen­yl)diazen­yl]-2-naphtholate ligands. The ligand anion exists in the *cis* form. The Na^I^ atom is coordinated by two phen­oxy O atoms from a neighboring Cr^III^ unit, two naphth­oxy O atoms from another neighboring Cr^III^ unit and two O atoms from acetone mol­ecules. The dinuclear complex forms a one-dimensional polymer running along the *c* axis.

## Related literature

For general background to charge-control agents, see: Tanaka (1995 ▶). For the preparation of the title compound, see: Yasumatsu *et al.* (2006 ▶). For related structures, see: Mizuguchi, Sato, Uta & Sato (2007 ▶); Mizuguchi *et al.* (2007*a* ▶,*b* ▶); Mizuguchi, Uta & Sato (2007 ▶); Sato *et al.* (2008 ▶).
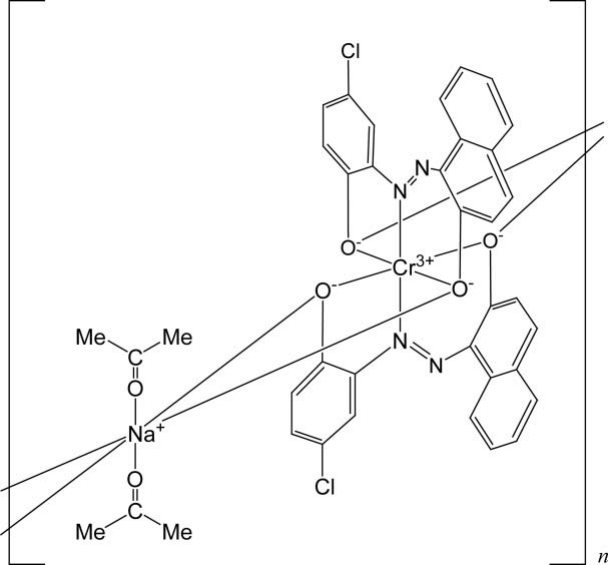


## Experimental

### 

#### Crystal data

[CrNa(C_16_H_9_ClN_2_O_2_)_2_(C_3_H_6_O)_2_]
*M* *_r_* = 784.55
Orthorhombic, 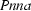

*a* = 18.5082 (17) Å
*b* = 26.199 (3) Å
*c* = 7.1726 (6) Å
*V* = 3478.0 (6) Å^3^
*Z* = 4
Mo *K*α radiation
μ = 0.55 mm^−1^
*T* = 93 K
0.26 × 0.10 × 0.09 mm

#### Data collection

Rigaku R-AXIS RAPID diffractometer
Absorption correction: multi-scan (**ABSCOR**; Higashi, 1995 ▶) *T*_min_ = 0.890, *T*_max_ = 0.947
22709 measured reflections
3314 independent reflections
1916 reflections with *I* > 2σ(*I*)
*R*_int_ = 0.153

#### Refinement

*R*[*F* ^2^ > 2σ(*F* ^2^)] = 0.110
*wR*(*F* ^2^) = 0.318
*S* = 1.03
3314 reflections
238 parameters
H-atom parameters constrained
Δρ_max_ = 2.03 e Å^−3^
Δρ_min_ = −0.59 e Å^−3^

### 

Data collection: *PROCESS-AUTO* (Rigaku, 1998 ▶); cell refinement: *PROCESS-AUTO*; data reduction: *CrystalStructure* (Rigaku/MSC, 2006 ▶); program(s) used to solve structure: *SIR2004* (Burla *et al.*, 2005 ▶); program(s) used to refine structure: *SHELXL97* (Sheldrick, 2008 ▶); molecular graphics: *ORTEPIII* (Burnett & Johnson, 1996 ▶); software used to prepare material for publication: *CrystalStructure*.

## Supplementary Material

Crystal structure: contains datablocks global, I. DOI: 10.1107/S1600536808000433/is2268sup1.cif

Structure factors: contains datablocks I. DOI: 10.1107/S1600536808000433/is2268Isup2.hkl

Additional supplementary materials:  crystallographic information; 3D view; checkCIF report

## Figures and Tables

**Table:** 

Cr1—O1	1.948 (4)
Cr1—O1^i^	1.948 (4)
Cr1—O2	1.993 (4)
Cr1—O2^i^	1.993 (4)
Cr1—N1	2.021 (5)
Cr1—N1^i^	2.021 (5)
Cl1—C1	1.752 (7)
Na1—O1	2.708 (4)
Na1—O1^ii^	2.708 (4)
Na1—O2^iii^	2.547 (4)
Na1—O2^i^	2.547 (4)
Na1—O3	2.274 (5)
Na1—O3^ii^	2.274 (5)

**Table:** 

O1—Cr1—O1^i^	91.84 (19)
O1—Cr1—O2	169.0 (2)
O1—Cr1—O2^i^	90.83 (18)
O1—Cr1—N1	87.12 (19)
O1—Cr1—N1^i^	90.86 (19)
O2—Cr1—O2^i^	88.56 (19)
O2—Cr1—N1	82.2 (2)
O2—Cr1—N1^i^	100.0 (2)
N1—Cr1—N1^i^	177.1 (2)
O1—Na1—O1^ii^	107.84 (18)
O1—Na1—O2^iii^	168.44 (17)
O1—Na1—O2^i^	64.50 (13)
O1—Na1—O3	82.45 (16)
O1—Na1—O3^ii^	91.90 (16)
O2^iii^—Na1—O2^i^	124.4 (2)
O2^iii^—Na1—O3	89.00 (17)
O2^iii^—Na1—O3^ii^	95.46 (17)
O3—Na1—O3^ii^	170.4 (2)
Cr1—O1—Na1	99.77 (18)
Cr1—O2—Na1^iv^	104.03 (19)

Symmetry codes: (i) 
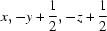
; (ii) 
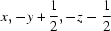
; (iii) 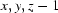; (iv) 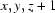.

**Fig. 1.**
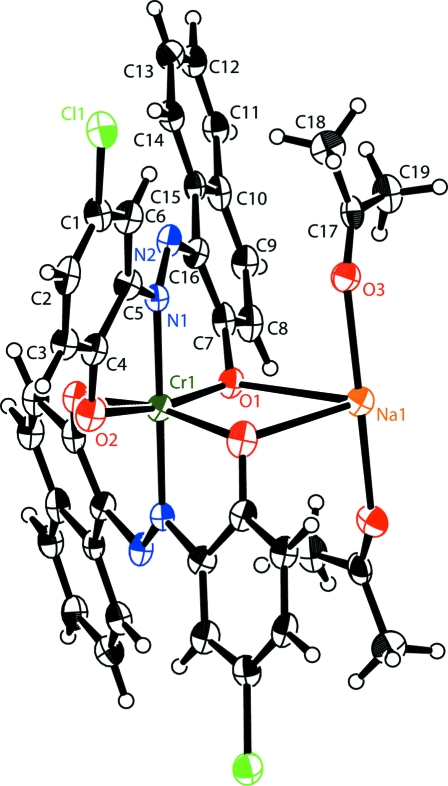
A view of the monomeric structure of the title compound, showing 50% displacement ellipsoids. Unlabelled atoms except for C17—C19 and O3 are related to labelled atoms by the symmetry operation (x, 1/2 - y, 1/2 - z), whereas those for C17—C19 and O3 by the symmetry operation (x, 1/2 - y, -1/2 - z).

**Fig. 2.**
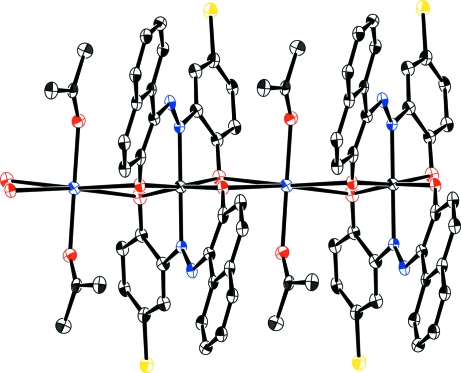
Polymeric structure of the title compound along the c axis.

## supplementary crystallographic information

### Comment

The title compound, (I), is an azo-Cr complex polymer that is used as a charge- control agent (CCA) of the negative type in electrophotography (Tanaka, 1995). The purpose of the investigation has been set out in our previous paper (Mizuguchi, Sato, Uta & Sato, 2007). We have previously reported the structure of a methanol-solvated azo-Fe complex with an ammonium cation (Mizuguchi *et al.*, 2007*a*) and its acetone solvate (Mizuguchi, Uta & Sato, 2007). Further structural analysis was carried out on the same azo-Fe complex but with a different cation, *i.e.* sodium one in place of the ammonium ion. The compound was recrystallized from an acetone (Mizuguchi *et al.*, 2007*b*) or acetonitrile solution (Sato, Uta & Mizuguchi, 2007). The present paper reports an azo-Cr complex polymer whose monomeric unit comprises an octahedral Cr^III^ unit and an octahedral Na^I^ one.

Figure 1 shows the *ORTEPIII* plot (Burnett & Johnson, 1996) of the monomeric unit of (I). The unit is composed of an octahedral Cr^III^ unit and an octahedral Na^I^ one. The anion complex exists in *cis*-form, just as in the ordinary azo-Fe complexes described in the above paragraph. Each Cr^III^ atom is chelated by the N and two O atoms from two [(5-chloro-2-oxidophenyl) diazenyl]-2-naphtholate ligands. On the other hand, the Na^I^ cation coordinates to a phenoxy O atom from the two ligands of each octahedral Cr^III^ unit, a naphthoxy O atom from the two ligands of each neighboring Cr^III^ unit and two acetone molecules. The octahedral Cr^III^ unit and the octahedral Na^I^ one are repeated alternately to form a one dimensional polymer along *c* through the four O ligands in common (Fig. 2). The polymer formation contributes to a significant stabilization of (I) whose property is pre-requisite for the CCA application in electrophotography.

### Experimental

The title compound was prepared according to the previously reported method (Yasumatsu *et al.*, 2006). Single crystals were recrystallized from an acetone solution. After 48 h, a number of single crystals were obtained in the form of blocks. Since the single crystals were found to include two solvent molecules according to the thermogravimetric analysis, reflection data were collected at 93 K.

### Refinement

All H atoms were placed in geometrically idealized positions (C—H = 0.93 or 0.96 Å) and constrained to ride on their parent atoms, with *U*_iso_ (H) = 1.2*U*_eq_(C) or 1.5*U*_eq_(methyl C). *R*-merge for the reflection data was 15.3%. This indicates poor crystal quality resulting in a rather high value of the *R* factor. The highest electron density (2.03 e Å^-3^) is located 0.64 Å from Cr1.

### Crystal data

**Table:**

[CrNa(C_16_H_9_ClN_2_O_2_)_2_(C_3_H_6_O)_2_]	*F*_000_ = 1612.00
*M**_r_* = 784.55	*D*_x_ = 1.498 Mg m^−^^3^
Orthorhombic, *P**n**n**a*	Mo *K*α radiation λ = 0.71075 Å
Hall symbol: -P 2a 2bc	Cell parameters from 14701 reflections
*a* = 18.5082 (17) Å	θ = 3.7–26.1º
*b* = 26.199 (3) Å	µ = 0.55 mm^−^^1^
*c* = 7.1726 (6) Å	*T* = 93 K
*V* = 3478.0 (6) Å^3^	Block, black
*Z* = 4	0.26 × 0.10 × 0.09 mm

### Data collection

**Table:**

Rigaku R-AXIS RAPID diffractometer	1916 reflections with *I* > 2σ(*I*)
Detector resolution: 10.00 pixels mm^-1^	*R*_int_ = 0.153
ω scans	θ_max_ = 26.0º
Absorption correction: multi-scan(*ABSCOR*; Higashi, 1995)	*h* = −22→22
*T*_min_ = 0.890, *T*_max_ = 0.947	*k* = −32→31
22709 measured reflections	*l* = −8→8
3314 independent reflections	

### Refinement

**Table:**

Refinement on *F*^2^	H-atom parameters constrained
*R*[*F*^2^ > 2σ(*F*^2^)] = 0.110	*w* = 1/[σ^2^(*F*_o_^2^) + (0.2*P*)^2^where *P* = (*F*_o_^2^ + 2*F*_c_^2^)/3
*wR*(*F*^2^) = 0.318	(Δ/σ)_max_ < 0.001
*S* = 1.04	Δρ_max_ = 2.03 e Å^−^^3^
3314 reflections	Δρ_min_ = −0.59 e Å^−^^3^
238 parameters	Extinction correction: none

### Special details

**Table:**

Geometry. All e.s.d.'s (except the e.s.d. in the dihedral angle between two l.s. planes) are estimated using the full covariance matrix. The cell e.s.d.'s are taken into account individually in the estimation of e.s.d.'s in distances, angles and torsion angles; correlations between e.s.d.'s in cell parameters are only used when they are defined by crystal symmetry. An approximate (isotropic) treatment of cell e.s.d.'s is used for estimating e.s.d.'s involving l.s. planes.
Refinement. Refinement of *F*^2^ against ALL reflections. The weighted *R*-factor *wR* and goodness of fit *S* are based on *F*^2^, conventional *R*-factors *R* are based on *F*, with *F* set to zero for negative *F*^2^. The threshold expression of *F*^2^ > σ(*F*^2^) is used only for calculating *R*-factors(gt) *etc*. and is not relevant to the choice of reflections for refinement. *R*-factors based on *F*^2^ are statistically about twice as large as those based on *F*, and *R*- factors based on ALL data will be even larger.

### Fractional atomic coordinates and isotropic or equivalent isotropic displacement parameters (Å^2^)

**Table:**

	*x*	*y*	*z*	*U*_iso_*/*U*_eq_	
Cr1	0.80893 (8)	0.2500	0.2500	0.0380 (5)	
Cl1	0.63892 (10)	0.02267 (8)	0.4102 (2)	0.0539 (6)	
Na1	0.7960 (2)	0.2500	−0.2500	0.0467 (9)	
O1	0.8822 (2)	0.24781 (18)	0.0550 (6)	0.0435 (11)	
O2	0.7318 (2)	0.2380 (2)	0.4390 (6)	0.0513 (12)	
O3	0.8062 (2)	0.1638 (2)	−0.2260 (6)	0.0512 (12)	
N1	0.8117 (2)	0.1729 (2)	0.2488 (6)	0.0351 (11)	
N2	0.8617 (2)	0.1418 (2)	0.2019 (6)	0.0407 (13)	
C1	0.6658 (3)	0.0867 (2)	0.4209 (8)	0.0462 (16)	
C2	0.6242 (3)	0.1212 (3)	0.5099 (8)	0.0461 (17)	
C3	0.6441 (3)	0.1722 (2)	0.5183 (9)	0.0437 (15)	
C4	0.7078 (3)	0.1896 (2)	0.4309 (8)	0.0407 (15)	
C5	0.7492 (3)	0.1516 (2)	0.3324 (9)	0.0426 (15)	
C6	0.7292 (3)	0.1006 (2)	0.3330 (9)	0.0435 (15)	
C7	0.9324 (3)	0.2119 (2)	0.0575 (7)	0.0396 (15)	
C8	1.0020 (3)	0.2267 (3)	−0.0206 (8)	0.0451 (15)	
C9	1.0569 (3)	0.1937 (2)	−0.0250 (8)	0.0461 (16)	
C10	1.0507 (3)	0.1422 (2)	0.0373 (7)	0.0434 (16)	
C11	1.1093 (3)	0.1072 (3)	0.0275 (8)	0.0458 (16)	
C12	1.1004 (3)	0.0574 (2)	0.0811 (8)	0.0462 (16)	
C13	1.0341 (3)	0.0416 (2)	0.1532 (8)	0.0457 (15)	
C14	0.9764 (3)	0.0742 (2)	0.1681 (8)	0.0445 (16)	
C15	0.9825 (3)	0.1249 (2)	0.1113 (7)	0.0371 (14)	
C16	0.9246 (3)	0.1624 (2)	0.1233 (7)	0.0385 (14)	
C17	0.8410 (4)	0.1242 (3)	−0.2542 (7)	0.0484 (17)	
C18	0.8125 (4)	0.0736 (3)	−0.1908 (11)	0.0583 (19)	
C19	0.9106 (3)	0.1238 (3)	−0.3583 (10)	0.0561 (19)	
H2	0.5815	0.1107	0.5665	0.055*	
H3	0.6151	0.1953	0.5824	0.052*	
H6	0.7580	0.0761	0.2753	0.052*	
H8	1.0082	0.2595	−0.0678	0.054*	
H9	1.1013	0.2048	−0.0706	0.055*	
H11	1.1540	0.1182	−0.0156	0.055*	
H12	1.1383	0.0343	0.0695	0.055*	
H13	1.0289	0.0080	0.1924	0.055*	
H14	0.9329	0.0624	0.2165	0.053*	
H18A	0.8513	0.0536	−0.1406	0.087*	
H18B	0.7913	0.0560	−0.2948	0.087*	
H18C	0.7764	0.0789	−0.0965	0.087*	
H19A	0.9490	0.1139	−0.2759	0.084*	
H19B	0.9202	0.1573	−0.4066	0.084*	
H19C	0.9076	0.0999	−0.4595	0.084*	

### Atomic displacement parameters (Å^2^)

**Table:**

	*U*^11^	*U*^22^	*U*^33^	*U*^12^	*U*^13^	*U*^23^
Cr1	0.0287 (8)	0.0449 (9)	0.0405 (8)	0.0000	0.0000	0.0033 (5)
Cl1	0.0448 (10)	0.0658 (13)	0.0511 (10)	−0.0180 (8)	0.0053 (7)	−0.0007 (7)
Na1	0.046 (2)	0.048 (2)	0.046 (2)	0.0000	0.0000	0.0035 (15)
O1	0.032 (2)	0.058 (3)	0.040 (2)	−0.001 (2)	−0.0023 (18)	0.0069 (19)
O2	0.024 (2)	0.077 (3)	0.053 (2)	0.007 (2)	−0.001 (2)	0.005 (2)
O3	0.045 (3)	0.056 (3)	0.052 (2)	0.006 (2)	−0.019 (2)	−0.002 (2)
N1	0.027 (2)	0.043 (3)	0.035 (2)	0.001 (2)	0.001 (2)	−0.001 (2)
N2	0.023 (2)	0.065 (4)	0.033 (2)	0.002 (2)	0.0005 (19)	0.007 (2)
C1	0.034 (3)	0.063 (4)	0.042 (3)	−0.009 (3)	−0.008 (2)	−0.001 (3)
C2	0.021 (3)	0.076 (5)	0.041 (3)	−0.017 (3)	−0.002 (2)	0.002 (3)
C3	0.021 (3)	0.059 (4)	0.051 (3)	0.005 (2)	0.001 (2)	0.012 (3)
C4	0.020 (2)	0.057 (4)	0.044 (3)	−0.004 (2)	−0.001 (2)	0.007 (2)
C5	0.023 (3)	0.055 (4)	0.050 (3)	−0.005 (2)	−0.008 (2)	0.006 (3)
C6	0.025 (3)	0.058 (4)	0.048 (3)	−0.001 (2)	0.002 (2)	0.005 (3)
C7	0.018 (2)	0.070 (4)	0.031 (2)	−0.002 (2)	−0.001 (2)	−0.003 (2)
C8	0.023 (3)	0.076 (4)	0.037 (3)	−0.002 (3)	−0.003 (2)	0.003 (3)
C9	0.027 (3)	0.071 (4)	0.040 (3)	0.003 (3)	−0.003 (2)	0.005 (3)
C10	0.018 (2)	0.080 (5)	0.032 (2)	−0.001 (2)	0.001 (2)	−0.002 (3)
C11	0.033 (3)	0.070 (5)	0.034 (3)	0.002 (3)	0.002 (2)	−0.004 (3)
C12	0.039 (3)	0.062 (4)	0.037 (3)	0.010 (3)	0.007 (2)	−0.003 (3)
C13	0.045 (4)	0.049 (4)	0.043 (3)	0.006 (3)	−0.008 (3)	−0.001 (3)
C14	0.032 (3)	0.060 (4)	0.041 (3)	0.006 (3)	−0.001 (2)	−0.003 (3)
C15	0.026 (3)	0.054 (4)	0.031 (2)	−0.005 (2)	0.002 (2)	−0.004 (2)
C16	0.022 (3)	0.057 (4)	0.037 (2)	−0.003 (2)	−0.004 (2)	0.008 (2)
C17	0.048 (4)	0.062 (5)	0.035 (3)	−0.002 (3)	−0.009 (2)	−0.007 (3)
C18	0.039 (4)	0.066 (5)	0.070 (4)	−0.007 (3)	0.010 (3)	0.007 (4)
C19	0.040 (4)	0.078 (5)	0.050 (3)	−0.013 (3)	0.000 (3)	−0.005 (3)

### Geometric parameters (Å, °)

**Table:**

Cr1—O1	1.948 (4)	C6—H6	0.930
Cr1—O1^i^	1.948 (4)	C7—C8	1.458 (8)
Cr1—O2	1.993 (4)	C7—C16	1.386 (9)
Cr1—O2^i^	1.993 (4)	C8—C9	1.336 (10)
Cr1—N1	2.021 (5)	C8—H8	0.930
Cr1—N1^i^	2.021 (5)	C9—C10	1.426 (10)
Cl1—C1	1.752 (7)	C9—H9	0.930
Na1—O1	2.708 (4)	C10—C11	1.421 (9)
Na1—O1^ii^	2.708 (4)	C10—C15	1.443 (8)
Na1—O2^iii^	2.547 (4)	C11—C12	1.371 (10)
Na1—O2^i^	2.547 (4)	C11—H11	0.930
Na1—O3	2.274 (5)	C12—C13	1.394 (9)
Na1—O3^ii^	2.274 (5)	C12—H12	0.930
O1—C7	1.323 (7)	C13—C14	1.372 (9)
O2—C4	1.345 (8)	C13—H13	0.930
O3—C17	1.238 (9)	C14—C15	1.393 (9)
N1—N2	1.278 (7)	C14—H14	0.930
N1—C5	1.418 (7)	C15—C16	1.456 (8)
N2—C16	1.403 (7)	C17—C18	1.497 (11)
C1—C2	1.349 (9)	C17—C19	1.488 (10)
C1—C6	1.381 (9)	C18—H18A	0.960
C2—C3	1.386 (10)	C18—H18B	0.960
C2—H2	0.930	C18—H18C	0.960
C3—C4	1.411 (8)	C19—H19A	0.960
C3—H3	0.930	C19—H19B	0.960
C4—C5	1.440 (9)	C19—H19C	0.960
C5—C6	1.387 (9)		
			
O1—Cr1—O1^i^	91.84 (19)	C3—C4—C5	116.0 (6)
O1—Cr1—O2	169.0 (2)	N1—C5—C4	111.7 (5)
O1—Cr1—O2^i^	90.83 (18)	N1—C5—C6	126.7 (5)
O1—Cr1—N1	87.12 (19)	C4—C5—C6	121.6 (5)
O1—Cr1—N1^i^	90.86 (19)	C1—C6—C5	118.7 (6)
O1^i^—Cr1—O2	90.83 (18)	C1—C6—H6	120.6
O1^i^—Cr1—O2^i^	169.0 (2)	C5—C6—H6	120.6
O1^i^—Cr1—N1	90.86 (19)	O1—C7—C8	115.1 (6)
O1^i^—Cr1—N1^i^	87.12 (19)	O1—C7—C16	126.7 (5)
O2—Cr1—O2^i^	88.56 (19)	C8—C7—C16	118.2 (5)
O2—Cr1—N1	82.2 (2)	C7—C8—C9	120.6 (7)
O2—Cr1—N1^i^	100.0 (2)	C7—C8—H8	119.7
O2^i^—Cr1—N1	100.0 (2)	C9—C8—H8	119.7
O2^i^—Cr1—N1^i^	82.2 (2)	C8—C9—C10	122.9 (6)
N1—Cr1—N1^i^	177.1 (2)	C8—C9—H9	118.5
O1—Na1—O1^ii^	107.84 (18)	C10—C9—H9	118.5
O1—Na1—O2^iii^	168.44 (17)	C9—C10—C11	122.2 (5)
O1—Na1—O2^i^	64.50 (13)	C9—C10—C15	118.9 (5)
O1—Na1—O3	82.45 (16)	C11—C10—C15	118.9 (6)
O1—Na1—O3^ii^	91.90 (16)	C10—C11—C12	120.6 (6)
O1^ii^—Na1—O2^iii^	64.50 (13)	C10—C11—H11	119.7
O1^ii^—Na1—O2^i^	168.44 (17)	C12—C11—H11	119.7
O1^ii^—Na1—O3	91.90 (16)	C11—C12—C13	119.5 (6)
O1^ii^—Na1—O3^ii^	82.45 (16)	C11—C12—H12	120.3
O2^iii^—Na1—O2^i^	124.4 (2)	C13—C12—H12	120.3
O2^iii^—Na1—O3	89.00 (17)	C12—C13—C14	121.9 (6)
O2^iii^—Na1—O3^ii^	95.46 (17)	C12—C13—H13	119.0
O2^i^—Na1—O3	95.46 (17)	C14—C13—H13	119.0
O2^i^—Na1—O3^ii^	89.00 (17)	C13—C14—C15	120.5 (6)
O3—Na1—O3^ii^	170.4 (2)	C13—C14—H14	119.7
Cr1—O1—Na1	99.77 (18)	C15—C14—H14	119.7
Cr1—O1—C7	120.0 (3)	C10—C15—C14	118.5 (5)
Na1—O1—C7	116.1 (3)	C10—C15—C16	116.9 (6)
Cr1—O2—Na1^iv^	104.03 (19)	C14—C15—C16	124.5 (5)
Cr1—O2—C4	110.9 (3)	N2—C16—C7	125.6 (5)
Na1^iv^—O2—C4	107.9 (3)	N2—C16—C15	112.0 (5)
Na1—O3—C17	149.7 (4)	C7—C16—C15	122.4 (5)
Cr1—N1—N2	131.1 (4)	O3—C17—C18	120.5 (6)
Cr1—N1—C5	111.7 (4)	O3—C17—C19	122.6 (7)
N2—N1—C5	116.8 (5)	C18—C17—C19	116.9 (6)
N1—N2—C16	117.4 (5)	C17—C18—H18A	109.5
Cl1—C1—C2	120.0 (5)	C17—C18—H18B	109.5
Cl1—C1—C6	118.3 (5)	C17—C18—H18C	109.5
C2—C1—C6	121.7 (6)	H18A—C18—H18B	109.5
C1—C2—C3	120.9 (6)	H18A—C18—H18C	109.5
C1—C2—H2	119.5	H18B—C18—H18C	109.5
C3—C2—H2	119.5	C17—C19—H19A	109.5
C2—C3—C4	120.9 (6)	C17—C19—H19B	109.5
C2—C3—H3	119.5	C17—C19—H19C	109.5
C4—C3—H3	119.5	H19A—C19—H19B	109.5
O2—C4—C3	124.2 (6)	H19A—C19—H19C	109.5
O2—C4—C5	119.8 (5)	H19B—C19—H19C	109.5
			
O1—Cr1—O1^i^—C7^i^	55.3 (4)	O3—Na1—O1^ii^—C7^ii^	−135.0 (4)
O1^i^—Cr1—O1—Na1	−176.84 (18)	O1^ii^—Na1—O3^ii^—C17^ii^	−80.9 (8)
O1^i^—Cr1—O1—C7	55.3 (4)	O3^ii^—Na1—O1^ii^—C7^ii^	37.2 (4)
O1—Cr1—O2—C4	−3.6 (12)	O2^iii^—Na1—O2^i^—Cr1	165.00 (18)
O1—Cr1—O2—Na1^iv^	112.2 (9)	O2^iii^—Na1—O2^i^—C4^i^	−77.2 (4)
O2—Cr1—O1—Na1	79.2 (10)	O2^i^—Na1—O2^iii^—Cr1^iii^	165.00 (18)
O2—Cr1—O1—C7	−48.7 (12)	O2^i^—Na1—O2^iii^—C4^iii^	−77.2 (4)
O1—Cr1—O2^i^—C4^i^	−107.6 (4)	O2^iii^—Na1—O3—C17	91.2 (8)
O1—Cr1—O2^i^—Na1	8.1 (2)	O3—Na1—O2^iii^—Cr1^iii^	−99.1 (2)
O2^i^—Cr1—O1—Na1	−7.5 (2)	O3—Na1—O2^iii^—C4^iii^	18.7 (3)
O2^i^—Cr1—O1—C7	−135.4 (4)	O2^iii^—Na1—O3^ii^—C17^ii^	−144.3 (8)
O1—Cr1—N1—N2	25.7 (4)	O3^ii^—Na1—O2^iii^—Cr1^iii^	72.5 (2)
O1—Cr1—N1—C5	−161.3 (3)	O3^ii^—Na1—O2^iii^—C4^iii^	−169.7 (3)
N1—Cr1—O1—Na1	92.39 (18)	O2^i^—Na1—O3—C17	−144.3 (8)
N1—Cr1—O1—C7	−35.5 (4)	O3—Na1—O2^i^—Cr1	72.5 (2)
O1—Cr1—N1^i^—N2^i^	−66.1 (4)	O3—Na1—O2^i^—C4^i^	−169.7 (3)
O1—Cr1—N1^i^—C5^i^	106.9 (3)	O2^i^—Na1—O3^ii^—C17^ii^	91.2 (8)
N1^i^—Cr1—O1—Na1	−89.70 (18)	O3^ii^—Na1—O2^i^—Cr1	−99.1 (2)
N1^i^—Cr1—O1—C7	142.4 (4)	O3^ii^—Na1—O2^i^—C4^i^	18.7 (3)
O1^i^—Cr1—O2—C4	−107.6 (4)	Cr1—O1—C7—C8	−148.7 (4)
O1^i^—Cr1—O2—Na1^iv^	8.1 (2)	Cr1—O1—C7—C16	32.3 (7)
O2—Cr1—O1^i^—C7^i^	−135.4 (4)	Na1—O1—C7—C8	91.2 (5)
O1^i^—Cr1—O2^i^—C4^i^	−3.6 (12)	Na1—O1—C7—C16	−87.7 (6)
O1^i^—Cr1—O2^i^—Na1	112.2 (9)	Cr1—O2—C4—C3	−166.3 (5)
O2^i^—Cr1—O1^i^—C7^i^	−48.7 (12)	Cr1—O2—C4—C5	15.4 (6)
O1^i^—Cr1—N1—N2	−66.1 (4)	Na1^iv^—O2—C4—C3	80.3 (6)
O1^i^—Cr1—N1—C5	106.9 (3)	Na1^iv^—O2—C4—C5	−98.0 (5)
N1—Cr1—O1^i^—C7^i^	142.4 (4)	Na1—O3—C17—C18	178.8 (6)
O1^i^—Cr1—N1^i^—N2^i^	25.7 (4)	Na1—O3—C17—C19	−3.9 (12)
O1^i^—Cr1—N1^i^—C5^i^	−161.3 (3)	Cr1—N1—N2—C16	−5.7 (7)
N1^i^—Cr1—O1^i^—C7^i^	−35.5 (4)	Cr1—N1—C5—C4	−12.3 (6)
O2—Cr1—O2^i^—C4^i^	83.4 (4)	Cr1—N1—C5—C6	170.9 (5)
O2—Cr1—O2^i^—Na1	−160.9 (2)	N2—N1—C5—C4	161.7 (5)
O2^i^—Cr1—O2—C4	83.4 (4)	N2—N1—C5—C6	−15.0 (8)
O2^i^—Cr1—O2—Na1^iv^	−160.9 (2)	C5—N1—N2—C16	−178.4 (4)
O2—Cr1—N1—N2	−156.8 (4)	N1—N2—C16—C7	−13.7 (8)
O2—Cr1—N1—C5	16.2 (3)	N1—N2—C16—C15	168.4 (4)
N1—Cr1—O2—C4	−16.9 (3)	Cl1—C1—C2—C3	−179.3 (4)
N1—Cr1—O2—Na1^iv^	98.9 (2)	Cl1—C1—C6—C5	176.7 (4)
O2—Cr1—N1^i^—N2^i^	116.1 (4)	C2—C1—C6—C5	−1.8 (9)
O2—Cr1—N1^i^—C5^i^	−71.0 (3)	C6—C1—C2—C3	−0.8 (9)
N1^i^—Cr1—O2—C4	165.2 (3)	C1—C2—C3—C4	1.2 (9)
N1^i^—Cr1—O2—Na1^iv^	−79.1 (2)	C2—C3—C4—O2	−177.5 (5)
O2^i^—Cr1—N1—N2	116.1 (4)	C2—C3—C4—C5	0.9 (9)
O2^i^—Cr1—N1—C5	−71.0 (3)	O2—C4—C5—N1	−2.0 (8)
N1—Cr1—O2^i^—C4^i^	165.2 (3)	O2—C4—C5—C6	175.0 (5)
N1—Cr1—O2^i^—Na1	−79.1 (2)	C3—C4—C5—N1	179.6 (5)
O2^i^—Cr1—N1^i^—N2^i^	−156.8 (4)	C3—C4—C5—C6	−3.5 (9)
O2^i^—Cr1—N1^i^—C5^i^	16.2 (3)	N1—C5—C6—C1	−179.6 (5)
N1^i^—Cr1—O2^i^—C4^i^	−16.9 (3)	C4—C5—C6—C1	4.0 (9)
N1^i^—Cr1—O2^i^—Na1	98.9 (2)	O1—C7—C8—C9	179.7 (5)
O1—Na1—O1^ii^—C7^ii^	−52.4 (4)	O1—C7—C16—N2	−0.9 (9)
O1^ii^—Na1—O1—Cr1	177.19 (16)	O1—C7—C16—C15	176.9 (5)
O1^ii^—Na1—O1—C7	−52.4 (4)	C8—C7—C16—N2	−179.8 (5)
O1—Na1—O2^iii^—Cr1^iii^	−56.8 (9)	C8—C7—C16—C15	−2.1 (8)
O1—Na1—O2^iii^—C4^iii^	61.0 (10)	C16—C7—C8—C9	−1.2 (8)
O2^iii^—Na1—O1—Cr1	−135.9 (8)	C7—C8—C9—C10	2.8 (9)
O2^iii^—Na1—O1—C7	−5.5 (10)	C8—C9—C10—C11	178.5 (6)
O1—Na1—O2^i^—Cr1	−6.48 (17)	C8—C9—C10—C15	−1.0 (9)
O1—Na1—O2^i^—C4^i^	111.3 (3)	C9—C10—C11—C12	−177.2 (5)
O2^i^—Na1—O1—Cr1	6.53 (17)	C9—C10—C15—C14	178.7 (5)
O2^i^—Na1—O1—C7	137.0 (4)	C9—C10—C15—C16	−2.1 (8)
O1—Na1—O3—C17	−80.9 (8)	C11—C10—C15—C14	−0.8 (8)
O3—Na1—O1—Cr1	−93.2 (2)	C11—C10—C15—C16	178.4 (5)
O3—Na1—O1—C7	37.2 (4)	C15—C10—C11—C12	2.3 (8)
O1—Na1—O3^ii^—C17^ii^	26.8 (8)	C10—C11—C12—C13	−2.8 (9)
O3^ii^—Na1—O1—Cr1	94.5 (2)	C11—C12—C13—C14	1.7 (9)
O3^ii^—Na1—O1—C7	−135.0 (4)	C12—C13—C14—C15	−0.2 (7)
O1^ii^—Na1—O2^iii^—Cr1^iii^	−6.48 (17)	C13—C14—C15—C10	−0.2 (7)
O1^ii^—Na1—O2^iii^—C4^iii^	111.3 (3)	C13—C14—C15—C16	−179.3 (5)
O2^iii^—Na1—O1^ii^—C7^ii^	137.0 (4)	C10—C15—C16—N2	−178.3 (4)
O1^ii^—Na1—O2^i^—Cr1	−56.8 (9)	C10—C15—C16—C7	3.7 (8)
O1^ii^—Na1—O2^i^—C4^i^	61.0 (9)	C14—C15—C16—N2	0.8 (8)
O2^i^—Na1—O1^ii^—C7^ii^	−5.5 (10)	C14—C15—C16—C7	−177.2 (5)
O1^ii^—Na1—O3—C17	26.8 (8)		

Symmetry codes: (i) *x*, −*y*+1/2, −*z*+1/2; (ii) *x*, −*y*+1/2, −*z*−1/2; (iii) *x*, *y*, *z*−1; (iv) *x*, *y*, *z*+1.
